# Social participation and mental health: moderating effects of gender, social role and rurality

**DOI:** 10.1186/1471-2458-13-701

**Published:** 2013-07-31

**Authors:** Daisuke Takagi, Katsunori Kondo, Ichiro Kawachi

**Affiliations:** 1 Graduate School of Medicine, The University of Tokyo, 7-3-1 Hongo, Bunkyo-ku, Tokyo 113-0033, Japan; 2 Center for Well-being and Society, Nihon Fukushi University, 5-22-35 Chiyoda, Naka-ku, Nagoya-shi, Aichi 460-0012, Japan; 3 Harvard School of Public Health, Harvard University, 677 Huntington Avenue, Boston, MA 02115, USA

**Keywords:** Japan, Social participation, Older people, Gender difference, Depressive symptoms, Key roles, Rural areas, Multilevel analysis

## Abstract

**Background:**

Previous studies have reported that older people’s social participation has positive effects on their health. However, some studies showed that the impacts of social participation on health differ by gender. We sought to examine whether the effects of social participation on mental health differ for men and women in a Japanese population. We also examined the moderating influence of social position within the organization as well as urban/rural locality.

**Methods:**

We used two waves of the Aichi Gerontological Evaluation Study’s longitudinal survey, which targeted residents with aged 65 years or over (*n* = 2,728) in a central part of Japan. The first wave survey was conducted in 2003, and the second wave in 2006. Depressive symptoms of the study participants were assessed using the short version of the Geriatric Depression Scale (GDS-15). A multilevel logistic regression model was used with individual-level as level 1 and the school district-level as level 2.

**Results:**

We found that higher social participation and performing key roles in the organization had protective effects on depressive symptoms for women. However, there were no main effects of these variables for the mental health of men. We found an interaction between social participation, organizational position, and rural residence among men only. That is, men who occupied leadership positions in organizations reported better mental health, but only in rural areas.

**Conclusions:**

Our findings support the notion that increasing the opportunities for social participation improves older people’s heath, especially for women. However, in the rural Japanese context, offering men meaningful roles within organizations may be important.

## Background

Studies from the field of gerontology have reported that older people’s social participation is associated with higher life satisfaction, higher self-esteem, lower rates of admission to a nursing homes, as well as lower mortality [1-3]. Generally, it is thought that social participation contributes to health by providing a sense of meaning in people’s lives as well as increasing access to social support [4]. For example, volunteer activities improve mental health by increasing the participants’ range of social networks, as well as their social prestige, access to resources and emotional gratification [5]. People’s social relationships formed by social participation lead to fulfillment of attachment, esteem, social approval, belongingness, social identity and security. Antonucci & Jackson [6] and Bandura [7] stated that such social relationships are useful because they are helpful to develop self-efficacy.

Some studies have reported that the effects of social participation vary depending on people’s background social characteristics. For example, previous reports found that social participation produces bigger benefits for the health of women than for men. Kavanagh et al. [8], for example, examined whether the effect of neighborhood-level political participation on self rated health differs according on gender. They found that neighborhood-level political participation enhanced women’s self rated health, but had no significant effect on men’s health. Spanish research on older people also showed that friendship connections are associated with a protective effect on cognitive impairment for women, but not for men [9]. A gender difference in the health effects of religious participation has also been reported. Norton et al. [10] reported that frequent attendance at church is associated with low prevalence of depressive symptoms among women, but it is associated in the opposite direction (i.e. with higher prevalence of depression) among men.

In contrast to the foregoing studies, some findings indicate that women can be negatively affected by social participation. Under certain circumstances, high social participation can contribute to higher psychological distress for women because of “role strain” associated with the duty of providing support to others [4]. Strazdins & Broom [11] refer to the gendered norms regarding the duty to provide “emotional labor” within social relations.

Based on the concept of role strain, Morrow-Howell et al. [12] examined the interaction effects of gender and participation in volunteer activities on self rated health, functional dependency and depressive symptoms, using panel data from three time points in the U.S. Although their results suggested that participation in volunteer activities positively affects the three health outcomes, none of the interaction effects between gender and participation were statistically significant. It is possible that there is a socially optimal level of social participation. Musick et al. [13] pointed out that both too little participation and too frequent participation in volunteer activities are more likely to be associated with negative impacts on the health of older people.

In the present study, we sought to address the question of whether the health effects of social participation vary by gender in a Japanese population. In addition, we sought to examine whether the gender difference in the effect of social participation has further interaction effects with other factors. First, the effect of social participation on health may vary according to the position occupied by the individual within the organization in which she/he participates. The theory of role strain suggests that it is more emotionally taxing when people assume positions of responsibility over others in the organization. Generally, because the role strain and emotional labor tend to be a burden for women [4,11], we predict that the interaction effect of social participation and performing key roles is stronger for women’s mental health than for men’s.

A second possible factor relevant for the health effects of social participation is urban/rural location. Personal networks in rural areas are more exclusive and closer than those in urban areas. In Japanese rural areas, the predominantly agricultural mode of production (i.e. rice farming) ensures that social bonds are tightly knit. For centuries, rice farmers in rural communities have developed norms of cooperation regarding water allocation (for irrigation of rice paddies) and mutual assistance during times of planting/harvesting. While highly functional in the sphere of production, these closely-knit communities may also cramp people’s freedom by producing sanctions against non-conformists. In places where such bonding networks exist, negative externalities such as intolerance are likely to be produced. Portes [14] pointed out the “excess claims on group members” as one of the downsides of social capital. Under such circumstances, over-involvement in the community (via social participation) may actually be a burden on people’s mental health. In Japan, the proportion of older people is much higher in rural areas than in urban areas, and tight bonding capital is also more likely to be observed in such areas. Thus, we were interested in examining the interaction effects between social participation and rural location in the Japanese context.

To our knowledge, there is little research that examines the interaction effect of older people’s social participation, role in organizations, and the urban/rural context of community on their mental health. Thus, the present study contributes to theoretical aspect of this field by revealing the complex interaction effect and develops practical aspect by suggesting the different manner of older people’s social participation according to sex and community characteristics.

## Methods

### Data

Data from the Aichi Gerontological Evaluation Study (AGES) project [15] were used for this study. The present study used longitudinal data from two time points. First, in 2003, investigators for the AGES project mailed a postal survey to 23,152 residents over the age of 65 years residing in 5 municipalities in Aichi Prefecture, Japan (The prefectures of Japan are the country’s 47 subnational jurisdictions. Prefectures are government bodies larger than cities, towns and villages – comparable of the states in the U.S.). In one of the five municipalities, simple random sampling (*n* = 5,000) was carried out from the Census of all individual aged over 65 years. In the remaining four municipalities, all residents aged 65 or older were sampled (i.e. a census was taken). The participation rate across the five sites was 52.0%, which is comparable to community-based surveys of this type.

In 2006, the investigators conducted the second wave survey based on the baseline respondents. In the second wave survey, the investigators mailed a postal survey to 11,991 residents who responded to the first survey. The response rate was 65.5% (*n* = 7,855). Our study protocol and informed consent procedure were approved by the Ethics Committee of Nihon Fukushi University.

In the analysis, respondents with missing values in sex, age, annual household income, the number of family members, years of education, marital status, social participation, roles in the organizations, and the Geriatric Depression Scale were omitted. 3,477 respondents were omitted because they were missing values for the above variables. In addition, in order to analyze the incidence (i.e. new occurrence) of depressive symptoms from 2003 to 2006, respondents who already had depressive symptoms in 2003 were omitted (i.e. 1,650 respondents were excluded due to depressive symptoms at baseline). As a result, the number of individual observations used in the analysis was 2,728 (Men = 1,541, Women = 1,187).

We conducted a multilevel regression analysis to take account of neighborhood-level contexts. The spatial unit in our multilevel analysis was elementary school district, of which there were 31 in our dataset. The school district-level data of this project was prepared by Hanibuchi [16]. For Japan as a whole, there are 19,672 school districts, and the average area of each school district is 16.86 km^2^ (SD = 38.59). In our dataset each district has an average area of 6.34 km^2^ (SD = 3.86). In Japan, a school district is defined as the primary residential spatial unit of people because local residents’ communities, senior citizens clubs, sports clubs etc. are organized within each district. Generally, a school district represents a geographical scale in which the elderly can travel easily by foot or bicycle. Thus, the present study used the school district as a proxy for the neighborhoods of people in our sample. The average number of respondents used in analysis per school district was 88.

### Measurements

#### Demographic variables

Sex of respondents, age, annual household income, the number of family members, years of education, and marital status were included as sociodemographic covariates. For annual household income, respondents were asked to identify their income level from the 14 predetermined categories (1 = less than 500 thousand yen, 2 = 500 thousand - 1 million yen, 3 = 1–1.5 million yen, 4 = 1.5 - 2 million yen, 5 = 2–2.5 million yen, 6 = 2.5 - 3 million yen, 7 = 3–4 million yen, 8 = 4–5 million yen, 9 = 5–6 million yen, 10 = 6–7 million yen, 11 = 7–8 million yen, 12 = 8–9 million yen, 13 = 9–10 million yen, 14 = more than 10 million yen). Using the annual household income and the number of family members, we created a variable of equivalised annual income, dividing the household income by the square root of the number of family members.

Years of education was obtained from responses to the question asking them to mark one response out from 4 categories (1 = less than 6 years, 2 = 6–9 years, 3 = 10–12 years, 4 = more than 13 years).

For marital status, respondents who were married and their spouse was alive at the time of survey were coded as 1 otherwise coded as 0.

All of these variables used in the analysis were responses from the first wave survey.

#### Social participation and key roles

Social participation was assessed by asking whether the respondents participated in the following 8 types of group in their neighborhood: Political group, Industry group / Trade association, Volunteer group, Civic / Consumer movement group, Religious group, Sport group, Neighborhood association / Senior citizens’ club, and Hobby group. Responses to each item was binarized (i.e., respondents who participated in the group was coded as 1 otherwise coded as 0 for each item). We chose not to create an index of participation by summing the groups because the alpha coefficient of the combined items was low (0.521 for Kuder-Richardson’s KR20). Thus, the present study used the 1st principal component score of these items as the social participation index variable. Table 1 shows a summary of the principal component analysis. We used the tetrachoric correlation matrix in this analysis because these items were binary.

**Table 1:** Summary of the principal component analysis

	**Factor loading**	**Communality**
	**1st principal component**	
Political group	.641	.410
Industry group/ Trade association	.480	.231
Volunteer group	.760	.578
Civic/Consumer movement group	.803	.644
Religious group	.368	.135
Sport group	.556	.309
Neighborhood associaiton/Senior citizens’club	.582	.338
Hobby group	.602	.363
Eigenvalue	3.001	
Contribution	37.600	

After asking the social participation question, we asked respondents whether they have occupied key roles within the organization such as president, facilitator or treasurer. Respondents responded in the affirmative were coded as 1 otherwise coded as 0.

For the variables of social participation and key role, we used the measurements from the baseline wave survey.

#### Outcome variable

We used depressive symptoms as our health outcome. This variable was measured both at the baseline survey in 2003 and the second wave survey in 2006. Depressive symptoms were measured by the short version of the Geriatric Depression Scale (GDS-15), using simple yes/no format, suitable for self-administration [17]. We used a cut-off point of 5 or above to indicate crassness of depressive symptoms. Previous validation studies of the short form GDS have reported that the sensitivity for clinical depression using a cut-off point of 5 ranges from 80% to 100%, while the specificity ranges from 56% to 90.5%. Therefore, the studies have concluded that a cut-off point of 5 was appropriate for the use of GDS as a screening tool for depression among community-dwelling elderly in the U.S. and Japan [17-20].

The present study used responses from respondents who did not report depressive symptoms at the first wave survey. That is, respondents who reported newly developed depressive symptoms from 2003 to 2006 were coded as 1, and respondents who remained symptom-free were coded as 0.

#### Neighborhood-level urban/rural location

We predicted that neighborhood rural location affects the effect of social participation on health. Thus, in order to create a cross-level interaction term, we used the school district-level proportion of workers engaged in primary industry (agriculture), following Hanibuchi [16] who used the primary industrial workers rate as one of indicators of rurality.

### Statistical analysis

On average, 88 respondents were nested within 31 neighborhoods (school district). A multilevel logistic regression model was used with individual-level as level 1 and the school district-level as level 2. The software used was HLM6.

To examine whether there were gender differences in the relationship between social participation and depressive symptoms, we stratified the analyses by gender. In the analysis, we included the interaction term of social participation (centered on the mean) and key role in order to examine how social participation with key roles affects mental health. In addition, to test whether neighborhood rural location moderates the association between social participation and health, we included a third-order cross-level interaction term, individual-level social participation x key roles x neighborhood rural location.

## Results

Table 2 contains the descriptive statistics for individual-level variables and neighborhood rural location.

**Table 2:** Descriptive statistics

	**Men (n = 1,541)**		**Women (n = 1,187)**	
	**n**	**%**	**n**	**%**
Age				
65-69	707	45.9	539	45.4
70-74	491	31.9	337	28.4
75-79	237	15.4	205	17.3
≥80	106	6.9	106	8.9
Equivalised annual income (million yen)				
<1.50	200	13.0	246	20.7
1.50-1.99	290	18.8	187	15.8
2.00-2.49	373	24.2	251	21.1
2.50-2.99	105	6.8	89	7.5
3.00-3.49	232	15.1	141	11.9
3.50-3.99	136	8.8	102	8.6
4.00-4.49	80	5.2	73	6.1
4.50-4.99	45	2.9	42	3.5
≥5.00	80	5.2	56	4.7
Years of education				
<6	19	1.2	39	3.3
6-9	728	47.2	621	52.3
10-12	507	32.9	441	37.2
≥13	287	18.6	86	7.2
GDS_15				
Depressive symptoms (≧5)	196	12.7	185	15.6
Non-depressed (≦4)	1345	87.3	1002	84.4
Marital status				
Married and spouse is alive	1450	94.1	767	64.6
Other	91	5.9	420	35.4
Role in social participation				
Having a certain key role	659	42.8	336	28.3
Other	882	57.2	851	71.7
	Mean	SD	Mean	SD
Social participation (0 - 8)^a^	1.82	1.45	1.72	1.37
*Neighborhood-level variable*				
	Mean		SD	
Rate of workers engaged in primary industry	11.56		10.98	

^a^ Social participation shown in this table is not the 1st principal component score but the simple sum of 8 items.

For the percentage of depressive symptoms, we found no major difference between men and women. For equivalised annual income, the proportion of respondents who have an income less than 150 million yen is slightly higher for women than for men. In addition the percentage of respondents with 6 or fewer years of education is also slightly higher for women than for men. The proportion of respondents who are married and living with a spouse is higher for men than for women. The proportion of respondents who report playing a key role in organizations is also higher for men than for women.

Table 3 shows the main effects of social participation and key role in the organization on depressive symptoms, as well as the interaction terms.

**Table 3:** Multilevel logistic regression of depressive symptoms and social participation, key role and rate of primary industrial workers for men and women

**Independent variables**	**Men**		**Women**	
	**Odds ratio**	**95%CI**	**Odds radio**	**95%CI**
*Individual-level variables*				
Intercept	0.03 ***	(0.01-0.10)	0.11 ***	(0.00-0.01)
Age				
65-69	Reference		Reference	
70-74	0.89	(0.60-1.31)	1.40 *	(1.02-1.92)
75-79	1.30	(0.75-2.25)	1.71 **	(1.21-2.41)
80 or older	0.88	(0.52-1.50)	2.50 ***	(1.56-4.01)
Equivalised annual income (million yen)				
<1.50	3.14 *	(1.08-9.13)	0.71	(0.35-1.43)
1.50-1.99	1.55	(0.55-4.37)	0.73	(0.38-1.39)
2.00-2.49	1.81	(0.71-4.60)	0.69	(0.31-1.56)
2.50-2.99	1.28	(0.41-3.97)	0.38 †	(0.13-1.09)
3.00-3.49	1.61	(0.56-4.58)	0.68	(0.28-1.67)
3.50-3.99	1.82	(0.46-7.18)	0.33 **	(0.16-0.68)
4.00-4.49	2.72 †	(0.87-8.55)	0.58	(0.21-1.63)
4.50-4.99	2.27	(0.71-7.19)	0.17 *	(0.03-0.86)
≥5.00	Reference		Reference	
Years of education				
<6	4.26 **	(1.53-11.87)	2.74 **	(1.37-5.49)
6-9	1.50 †	(0.97-2.31)	1.62	(0.88-2.99)
10-12	1.21	(0.82-1.78)	1.74 †	(0.94-3.23)
≥13	Reference		Reference	
Living with spouse	1.68 †	(0.92-3.10)	1.15	(0.90-1.46)
Social participation	0.76	(0.31-1.87)	0.53 *	(0.28-0.99)
Role in social participation	0.81	(0.60-1.10)	0.57 *	(0.37-0.88)
Social participation * Role	0.32 †	(0.09-1.16)	0.34	(0.06-1.81)
*Individual × Neighborhood*^*a*^				
Social participation * Role * Primary industry	0.28 ***	(0.16-0.50)	0.68	(0.16-2.89)
Random Effect	Variance		Variance	
	Component		Component	
Intercept	0.01		0.01	
Individual-level *n*	1541		1187	
Neighborhood-level *n*	31		31	

Note: ***p <.001, **p < .01, *p < .05, †*p* < .10.

^a^ Social participation and role are individual-level variables, and primary industry is a neighborhood-level variable.

First, Table 3 shows that social participation had a protective main effect on women’s depressive symptoms, while it was not significant for men (odds ratio: 0.53 for women (95% CI: 0.28-0.99), 0.76 for men (95% CI: 0.31-1.87)).

Second, and contrary to our expectation, performing a key role in the social organization had a protective main effect on women’s depressive symptoms, while it did not have a significant impact on men’s depression (odds ratio: 0.57 for women (95% CI: 0.37-0.88), 0.81 for men (95% CI: 0.60-1.10)).

Third, the interaction effect of social participation x key role was significant for men. That is, a protective effect on depressive symptoms was observed only for men who reported both high social participation and performing a key role in the organization. This interaction term was not statistically significant among women.

Fourth, Table 3 shows that the interaction effect of social participation and key roles are further enhanced for men residing in rural neighborhoods where the rate of primary industrial workers is high (odds ratio: 0.28 (95% CI: 0.16-0.50)). However, contrary to our prediction, the interaction effect on women’s mental health was not affected by the rural location of the neighborhood (odds ratio: 0.68 (95% CI: 0.16-2.89)).

**Figure 1:**
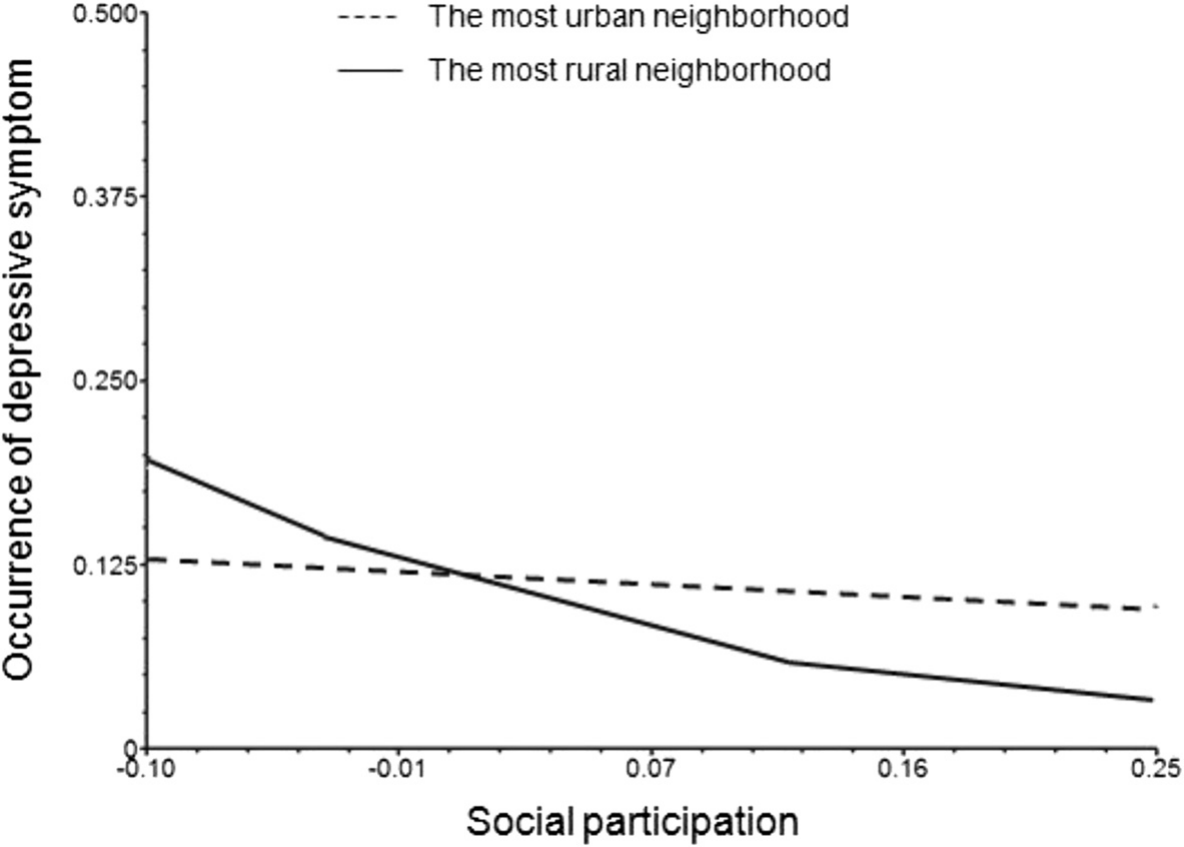
The interaction effect of social participation and neighbourhood rural location on depressive symptoms for MEN (men who occupy key roles in the organization).

**Figure 2:**
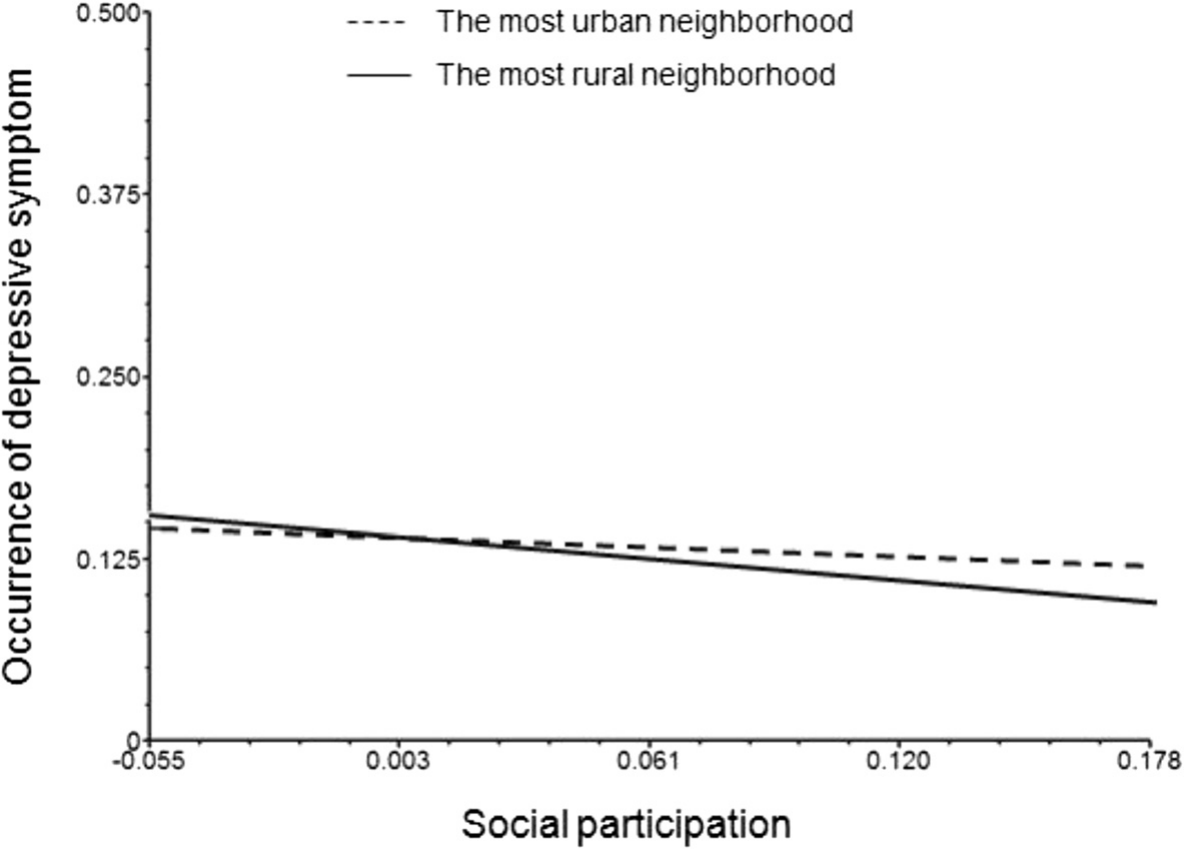
The interaction effect of social participation and neighbourhood rural location on depressive symptoms for WOMEN (women who occupy key roles in the organization).

For ease of presentation, Figures 1 and 2 show the predicted probabilities of depressive symptoms including the interaction effects. In the figures, the predicted probabilities are based on contrasting neighborhoods with the highest and lowest proportions of agricultural workers (i.e. most rural vs. most urban).

From Figure 1, it can be seen that for men who occupy key roles in the organization, higher social participation is associated with lower risk of depressive symptoms – and the association is stronger in more rural neighborhoods (i.e. neighborhoods with higher proportion of agricultural workers). The same trend is not observed in neighborhoods with low proportion of agricultural workers. By contrast, Figure 2 shows that among women there appears to be no difference in risk according to rural/urban location.

## Discussion

We summarize our main findings. First, the main effects of social participation and performing key roles in the participation were significant only for women. Contrary to our prediction that performing a role with responsibilities may have negative impacts on women’s health (from the perspective of role strain theory and emotional labor), the results of our study found the opposite. Thus, this result is consistent with Kavanagh et al.’s [8] and Norton et al.’s [10] arguments that women receive more benefit from their social participation than men. Because women are more likely than men to make close friends from their large and diverse networks [21], they may tend to receive positive benefits from social participation.

Our next notable finding was that among men there was an interaction effect between social participation and performance of key roles. In contrast to Kawachi and Berkman’s [4] argument that frequent social participation may bring about psychological distress for women, this suggests that social participation which provides the individual with a social role does not adversely impact the mental health of women, and may even promote the mental health of men. This also suggests that occupying a key position within an organization a necessary condition for men to receive positive effects from their social participation. The same interaction effect was not found for women’s depressive symptoms. In Japanese society, which is characterized by strong patriarchal values, males seek meaning and identity by being valued in the workplace (as opposed to in the home). This orientation may spill over into retirement, such that men feel rewarded by seeking positions of authority of responsibility within the social organizations in which they participate. The present study’s suggestion that it is important to have roles in social participation for retired men is critical for developing community-based interventions to promote the health of elders.

A third notable finding of our study is the cross-level interaction effect between social participation x performance of key roles x rural residence. As Figure 1 shows, this result suggests that the interaction effect of social participation and key roles on depressive symptoms is enhanced (for men) in rural areas, while the interaction effect is non-significant in urban areas. Although we predicted that closed networks are more likely to be formed in rural areas and that such networks might be associated with adverse effects on mental health, our results suggested the opposite – primarily boosting the mental health of men who perform key roles. In contrast to rural areas, open and mobile networks are more likely to be formed in urban areas, but the extent of cohesion in such networks tends to be low and the degree of members’ cooperation may also be low. In such a setting, performing key roles may add to mental burden. Our results offer new insight into health promotion among the elderly, and shed light on the interaction between the social context and social participation.

In addition, because our analyses focused on only new occurrence of depressive symptoms (i.e. respondents who already had depressive symptoms at baseline were excluded), the causal interpretation of our findings is strengthened.

Parallel with providing some novel insights into the theoretical aspects of older people’s social participation, our results also suggest some practical implications. In the health promotion literature, increasing the opportunities for social participation has been suggested as a means of improving older people’s health [22-24]. Our findings support that notion, but also suggest complexities based on gender.

Some limitations of our study should be noted. The present study used the proportion of agricultural workers as a proxy indicator of rural status. In addition, the characteristics of social network (tightly bonded) in Japanese rural areas described in this paper are speculative, and require further ethnographic elaboration. Ideally, instead of using urban/rural as the indicator, a more theoretically-grounded approach would have been to stratify the analyses based upon network characteristics such as size, closeness, openness and mobility. Future studies should measure such detailed characteristics and incorporate them into the analyses.

Second, a high number of respondents were omitted from our analyses because of missing values. Generally, missing responses tend to be higher among older people and in mail surveys. The missing responses may have biased the findings of our study.

## Conclusion

Our findings support the notion that increasing the opportunities for social participation improve older people’s health, especially for women, but also suggest nuances based on the social context (Japanese society), sociodemographic characteristics (gender), social position (whether occupying key roles within organizations), and location (rural vs. urban) – that is, in the rural Japanese context, offering men meaningful roles within organizations may be important.

## Competing interests

The authors declare that they have no competing interests.

## Authors’ contributions

DT performed the statistical analysis and drafted the manuscript. KK made substantial contributions to design and acquisition of data. IK revised the drafted manuscript critically for important intellectual content. All authors read and approved the final manuscript.

## Pre-publication history

The pre-publication history for this paper can be accessed here:

http://www.biomedcentral.com/1471-2458/13/701/prepub
